# Sleep Posture Detection via Embedded Machine Learning on a Reduced Set of Pressure Sensors

**DOI:** 10.3390/s25020458

**Published:** 2025-01-14

**Authors:** Giacomo Peruzzi, Alessandra Galli, Giada Giorgi, Alessandro Pozzebon

**Affiliations:** 1 Department of Information Engineering, University of Padova, 35122 Padova, Italy; giacomo.peruzzi@unipd.it (G.P.); giada.giorgi@unipd.it (G.G.); alessandro.pozzebon@unipd.it (A.P.); 2 Department of Electrical Engineering, Eindhoven University of Technology, 5612 AZ Eindhoven, The Netherlands

**Keywords:** artificial intelligence, support vector machine, pressure sensors, embedded machine learning, internet of things, sensor selection, sleep posture recognition, obstructive sleep apnea

## Abstract

Sleep posture is a key factor in assessing sleep quality, especially for individuals with Obstructive Sleep Apnea (OSA), where the sleeping position directly affects breathing patterns: the side position alleviates symptoms, while the supine position exacerbates them. Accurate detection of sleep posture is essential in assessing and improving sleep quality. Automatic sleep posture detection systems, both wearable and non-wearable, have been developed to assess sleep quality. However, wearable solutions can be intrusive and affect sleep, while non-wearable systems, such as camera-based approaches and pressure sensor arrays, often face challenges related to privacy, cost, and computational complexity. The system in this paper proposes a microcontroller-based approach exploiting the execution of an embedded machine learning (ML) model for posture classification. By locally processing data from a minimal set of pressure sensors, the system avoids the need to transmit raw data to remote units, making it lightweight and suitable for real-time applications. Our results demonstrate that this approach maintains high classification accuracy (i.e., 0.90 and 0.96 for the configurations with 6 and 15 sensors, respectively) while reducing both hardware and computational requirements.

## 1. Introduction

Sleep plays an important role in human life, as it enables recovery from physical and mental fatigue and the consolidation of memories [1]. For this reason, the quality of sleep is an essential aspect. Low-quality or inadequate sleep leads to impaired physical health and can cause cognitive and psychological problems. For example, people who do not sleep well are more prone to chronic diseases such as obesity, diabetes [2], and hypertension [3]. Inadequate sleep is also one of the causes of car accidents [4] and lower productivity [5].

In particular, Obstructive Sleep Apnea (OSA) is a syndrome characterized by a complete or partial blockage of the upper airway during sleep. Sleeping in the side position alleviates the disorder for patients with mild and moderate OSA [6], while the supine position has negative effects on breathing. It was demonstrated in [7] that the Apnea-Hypopnea Index (AHI) is higher in supine than in lateral posture. In the context of OSA, accurately determining whether a subject is in a supine position is essential for achieving a correct diagnosis and providing an appropriate, tailored treatment plan. For individuals with supine-related OSA—where sleep apnea occurs only when sleeping in the supine position—the condition can often be managed by simply avoiding this posture. In contrast, non-position-related OSA typically requires more invasive treatments, such as CPAP therapy or surgery. Therefore, accurately detecting the subject’s position is critical to prevent unnecessary invasive treatments and ensure effective management of the condition.

In recent years, several devices for the automatic recognition of sleep posture have been introduced. These devices can be roughly classified into two categories: wearable and non-wearable. For example, in [8] the authors propose measuring static acceleration using an accelerometer embedded into a smartwatch. This approach is based on the assumption that wrist position is closely correlated with body position. Features extracted from the acquired signals are then used to train a classifier for finally obtaining an estimated posture. Various features are extracted from the accelerometer signals, which are then used to train classifiers (i.e., Naive Bayes (NB), Bayesian Network (BN), Decision Tree (DT), and Random Forest (RF)) for recognizing sleep postures. The authors observed that RF distinguishes sleep postures with 91.7% accuracy and outperforms the other methods. Another example is provided in [9]: in this case, the accelerometers were placed on the chest and ankles, and the acquired data were exploited to train and test a Generalized Matrix Learning Vector Quantization (GMLVQ) classifier, a machine learning (ML) model similar to the k-Nearest Neighbours (kNN) algorithm, aimed at recognizing sleep posture. However, both these solutions present some drawbacks. First of all, acquired signals are usually affected by several motion artifacts that reduce the signal quality, requiring additional operations for denoising. Secondly, the usage of wearable devices can be perceived as too invasive by people while they sleep, thus worsening the quality of sleep, with the risk of inferring incorrect conclusions.

Methods based on cameras or sensors embedded in the bed belong to the category of non-wearable devices. The former can employ various types of cameras like common digital visible light cameras, infrared cameras, or Kinect cameras. Image processing and ML techniques are then applied to the acquired visual data in order to automatically recognize different postures [10,11]. Camera-based approaches are, in general, very expensive and light-sensitive, require installation, and violate the privacy of people being recorded [12]. Some of these limitations can be overcome with the use of sensors embedded in the bed or bed sheets. For instance, Li et al. [13] proposed a vibration-based sleep monitoring system, embedded in the bed frame, that includes not only the identification of sleep posture but also the estimation of vital parameters. Other methods are based on pressure sensors, such as the one proposed by Liu et al. [14] based on high-resolution pressure maps obtained from a dense array of textile electrodes integrated into bed sheets to monitor sleeping posture. Instead, Li et al. [15] embedded a capacitive pressure sensor array directly into the mattress. This system is constructed using dust-free cloth and sponge dielectric layers. The data from the sensor arrays are processed by using a Multi-Class Support Vector Machine (SVM) model to accurately identify four different sleeping postures. Matar et al. [16] propose an array of 64×27 piezoresistive pressure textile sensors placed under the subject’s bed sheet to collect data, feeding a supervised Neural Network (ANN) classification model. The same strategy with fewer sensors is employed in [12]. The system is formed by 171 sensors placed in a 19×9 grid structure that detects the distribution of body pressure on the mat during sleep. Then, through a Convolutional Neural Network (CNN) the collected data are analyzed, and different sleeping postures are recognized. These methods achieve high accuracy in posture classification. However, the production cost and computational complexity are considerable.

This work addresses the problem of recognizing supine and non-supine sleep postures. In particular, the sleep posture detection system proposed in this paper aims at significantly reducing the number of pressure sensors by using a sensor-selection algorithm based on an SVM. This leads to a double advantage: the possibility of using a very low-cost platform to collect pressure signals from the reduced set of selected sensors and the possibility of executing a lightened classification algorithm directly on a local resource-constrained device. Specifically, we employ an embedded ML model running on a microcontroller to detect sleep postures; this method aims at resorting to the data acquired from the sensors. Raw data are locally processed to obtain a posture estimation, thus avoiding their transmission towards remote units for the analysis.

The rest of this paper is structured as follows: at first, the system architecture is introduced in Section 2, while the dataset on which the ML models are trained and tested is presented in Section 3; it contains data gathered during an experiment involving volunteers lying on a pressure mattress with embedded pressure sensors. In Section 4, the ML models and their optimization are shown by explaining their design rationale. Finally, the results are presented and discussed (see Section 5), and some final remarks are reported (see Section 6).

## 2. System Architecture

Embedded systems are usually resource-constrained hardware platforms, thus implicitly entailing limitations that must be particularly considered whenever embedded ML models are designed and developed to be deployed on them. In this context, while ANNs running on embedded devices require particular hardware architectures in terms of memory and computational power, the ML models proposed in this work are designed to be deployed on simpler platforms featuring low-complexity architectures. Indeed, simple linear models were developed, which can be executed even on microcontrollers by performing a multiply–accumulate operation. The effectiveness of these solutions, if following a conservative approach aiming at reducing costs, energy consumption, and system complexity, is based on the definition of a reduced set of sensors.

In order to define the number of pressure sensors to be selected, we consider the constraints imposed by a typical embedded system: in this case, the total number of employable analog sensors is limited by the overall number of Analog-to-Digital Converter(ADC) channels. Low-power microcontrollers feature in general one or two eight-channel ADCs (configurations with three ADCs are also present, but they are not taken into account in this work since they are seen as more complex and usually not compliant with a low power requirement). This means that the number of sensors that can be used to acquire the signals without resorting to additional external components like multiplexers should be lower than 8 or 16.

Following these assumptions, two different system configurations are assumed in this work: a “sparse” one, compliant with the presence of a single ADC, and a “dense” one, used for the case with two ADCs. The “sparse” configuration foresees the selection of six pressure sensors: this layout was chosen considering as a reference the Arduino UNO board, one of the most widespread prototyping boards, which is provided with six ADC inputs. We chose this value since it can be seen as more challenging with respect to the usage of all eight ADC channels while allowing at the same time the actual implementation of the system on a widely employed single-board microcontroller platform.

The “dense” configuration was shaped on the two ADC configuration; it is present, for example, in the widely employed Arduino Mega board. In this case, we opted for a total number of 15 pressure sensors: this number was chosen in order to fully exploit the available channels while keeping one input channel available for connecting any other sensor (e.g., a temperature sensor). Obviously, in this case too, alternative choices may have been made. However, the aim of these two configurations was to demonstrate the effectiveness of the proposed solution with a total number of sensors lower than the number of available ADC channels, and the two configurations are thus perfectly compliant with such a requirement.

## 3. Dataset

The posture estimator is based on an ML algorithm capable of fusing information collected from many pressure sensors, thus obtaining high-level information concerning the posture assumed by a subject during sleep. For this purpose, we consider the data collected by Pouyan et al., publicly available from an online database [17]. Data were collected using the Vista Medical SoftFlex 2048 mattress produced by Force Sensitive Application (FSA), a light, thin, and flexible pressure mat covering a queen-size regular mattress. Such a typology of sensing structure, directly positioned above the mattress, allows the removal of any variability in the collected data deriving from differences in the typology of the mattress; indeed, the sensors are directly placed below the body and above the mattress, integrated in the mat, thus maintaining their arrangement regardless of the mattress below them. Data refer to normalized pressure values, ranging from 0 to 1000, collected from a matrix of uniformly spaced pressure sensors. Pressure sensors are arranged according to a 32×64 uniform grid, for a total of 2048 sensors. The dataset contains observations obtained for 13 different subjects in different positions, namely, left side (L), right side (R), and supine (S).

For what concerns the representativeness of the data, the dataset provides good variability regarding body size, with height values ranging from 169 cm to 186 cm and body weights with values ranging from 66 kg to 100 kg (see Table 1 for the detailed morphological information of the subjects.). For each possible posture, pressure values were collected with a sampling frequency of 1 Hz and over observation windows with an average duration of 120 s. During the whole observation interval, the posture of a given subject remains almost the same, with a variability that mainly depends on slight physiological movements (e.g., leg movements). Transitions from a given posture to a different one are not provided in the dataset. For this reason, the algorithm discussed in this paper only focuses on the part concerning the design of the classifier for automatically labeling the posture assumed by a monitored subject when in stationary, or almost stationary, positions.

The first operation conducted on the original dataset consisted of discarding meaningless data. In fact, the original dataset was collected to reproduce typical hospital settings, also containing observations where wedges were placed on the right/left side of the subject and others collected by tilting the bed. Since the purpose of our work consists instead of developing a home monitoring system, we discarded these observations from the database.

A second operation concerned instead a suitable re-labeling of the data. Observations provided in the original dataset were classified by considering three different postures: L, R, and S, as previously explained. However, because the target of the proposed system consists of detecting postures potentially causing OSA, observations were split into only two classes, i.e., “Supine” (S) and “No-Supine” (NS). The former includes observations originally labeled as S, while the second contains the observations originally labeled as L and R. The resulting dataset contains 52 observations labeled as NS, respectively, 26 L and 26 R, and 78 observations labeled as S. Then, the dataset was split into training and test sets: the former includes observations related to 10 subjects, thus counting 40 observations labeled as NS (20 L and 20 R, in turn) and 60 observations labeled as S, while the latter comprises the observations of the other 3, meaning that it has 12 observations labeled as NS (6 L and 6 R, in turn) and 18 observations labeled as S.

## 4. Sensor Fusion

In this section, we describe the algorithm employed for combining data obtained from a given number of selected pressure sensors in order to estimate the posture of the monitored subject by considering only two possible outcomes: *supine* and *no-supine*, useful to detect the risk of OSA. For this purpose, a very simple linear SVM algorithm will be exploited, characterized by low computational complexity.

### 4.1. Preprocessing Phase

**Figure 1:**
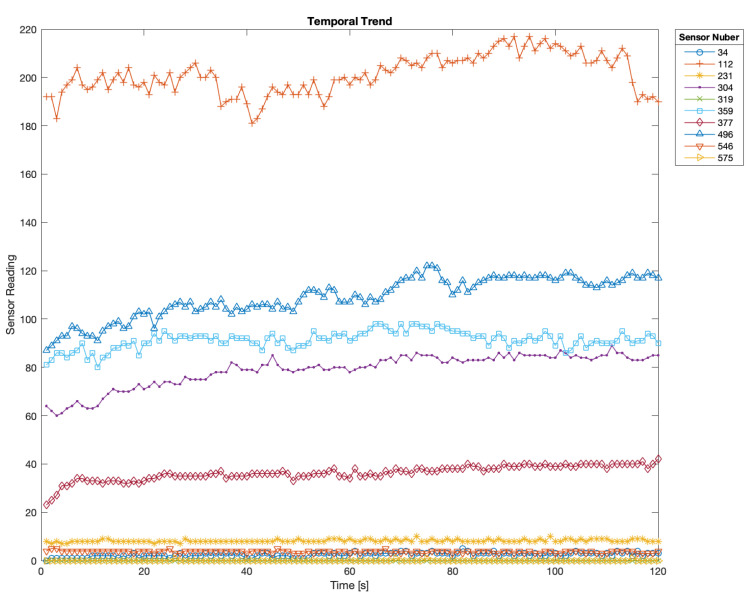
Temporal trend of some of the pressure sensors forming the mattress. Such readings are relative to a subject in supine posture, but other subjects in other postures provide similar results. Reading values remain almost constant throughout the acquisition timespan, or marginally vary, thus making the median ideal for data preprocessing.

The signal from each sensor is sampled at a frequency of 1 Hz; however, detecting the subject’s position every second is both redundant and unnecessarily resource-intensive. To optimize computational efficiency, we aggregate the data from the i-th time series (corresponding to the i-th sensor) by calculating its median value. The median was chosen over other metrics (e.g., mean and mode) because of its robustness against potential outliers and artifacts, ensuring more reliable data representation. Such a choice is also validated according to Figure 1: it displays the temporal trend of the readings of some pressure sensors during the whole acquisition for a given subject in supine position (other positions provide similar results), showing that such readings values slightly vary, or even remain almost constant. Moreover, Figure 1 also provides hints on how to deal with movement detection. Indeed, it can be detected by carrying out a stationariness test, and in the case of negative results by avoiding performing the posture prediction, since it would be unreliable.

### 4.2. Linear Support Vector Machine

SVMs are supervised ML models that set up a hyperplane in a high-dimensional space, which can be used for classification, regression, or other tasks. Qualitatively, better performances are achieved by the hyperplane with the largest distance to the nearest training datapoints of any class (the so-called support vectors), because the larger the margin the lower the generalization error of the classifier.

The training phase of a linear SVM is formulated as follows. Given a training set formed by datapoints $x_{i}\in R^{p},i=1,\ldots ,n$ with *p* features (i.e., the dimensionality of the training set), whose $x_{i}$ are split into two classes, in this specific problem such classes, respectively, code the “Supine” (S) and “No-Supine” (NS) classes (in turn representing the relative posture), with the labels $y\in {\left\{1,-1\right\}}^{n}$; the SVM aims at finding $w\in R^{p}$ (i.e., the coefficient vector) and b∈R (i.e., the bias) in order to maximize the number of correct predictions, given by $sign(w^{T}x+b)$. This translates into the following optimization problem:

(1) $$\begin{array}{cc} \min_{w,b}\; & {\left||w|\right|}_{j}+C\sum_{i=1}^{n}\max(0,1-y_{i}\left(w^{T}x_{i}+b\right))\\ s.t.\; & y_{i}\left(w^{T}x_{i}+b\right)\geq 1-\xi_{i},\\ \; & \xi_{i}\geq 0,i=1,\ldots ,n \end{array}$$

where j=1,2, thus, respectively, imposing the minimization of the l1 or l2 norm of *w*, in turn leading to a sparse, or non-sparse, *w* vector (according to the kind of problem needing to be solved); *C* is the regularization parameter and $\xi_{i}$ is the distance of datapoint $x_{i}$ from the separating hyperplane. The value of *C* determines the strength of the regularization: the higher the parameters, the less the regularization. In other words, high values of *C* lead to a model trying to fit the training set as best as possible, but overfitting may likely occur; conversely, low values of *C* result in a model whose coefficient vector *w* is close to zero. Moreover, when the l1 norm is minimized, vector *w* turns out to be sparse (the lower the value of *C*, the higher the number of null elements in vector *w*), meaning that an implicit selection of the features is accomplished during the training phase.

### 4.3. Sensor Selection

The first step to design a low-cost embedded sensorized system consists of choosing a subset of the most informative sensors. Different strategies can be adopted to select a given number of sensors. This operation corresponds to spatial down-sampling, where the related problem consists of choosing the sensors on the grid—number and positions—that allow better discrimination between the supine position and the other one.

In this paper, we consider two different strategies to perform this down-sampling.

**Figure 2:**
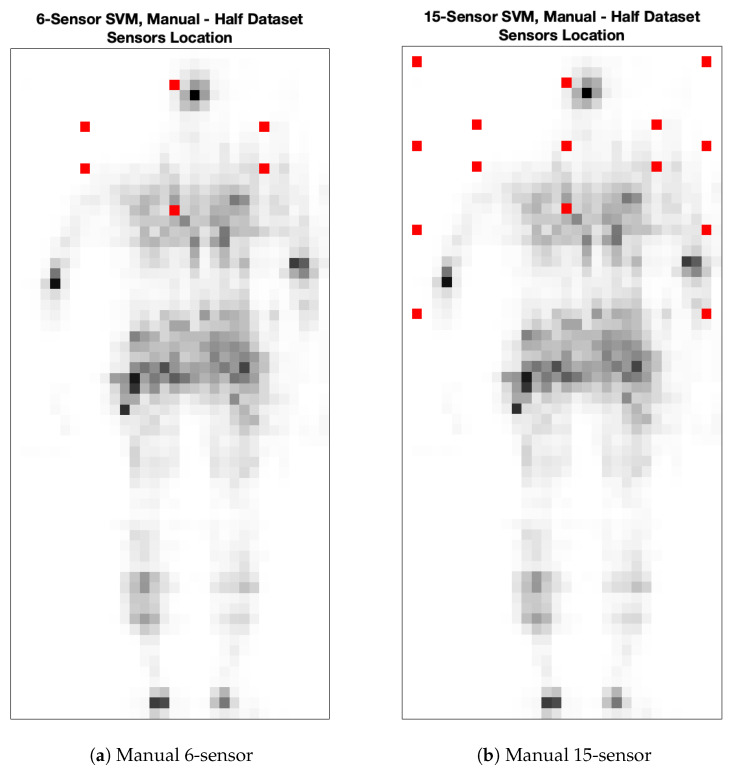
Selected sensors are indicated by red dots. Gray-scale dots represent an example of pressure values acquired by the high-density sensor grid that occupies the whole rectangle. The shape is an example of a pressure field measured by the original grid.

- The former consists of automatically selecting the most significant sensors through the procedure of minimization, specifically exploiting the l1 norm in Equation (1), implemented during the training of an SVM classifier. This approach is applied either on the full original sensor grid or only on the upper part of the grid, which is less affected by leg movements.
- The second approach consists instead of manually selecting a given subset of sensors by following an empirical approach, based on the human shape. An example is reported in Figure 2. The selected sensors are represented by red dots: in the left figure, we choose an elliptic configuration that covers the cervical–lumbar region, while on the right we choose a more dense configuration, where some sensors have been placed also on the side regions. Obviously, other configurations can be chosen. However, in this paper, the attention is mainly focused on comparing the accuracy achievable with automatic selection with the accuracy that could be achieved instead by manually selecting the sensors.

A possible advantage of this manual selection consists of a simpler configuration (in this paper, we chose a symmetric configuration) that could reduce the production cost. The former solutions based on automatic selection would instead require a precise positioning of the sensors during the production stage.

### 4.4. Hyperparameter Tuning

As was explained in Section 4.2, the regularization parameter *C* plays a key role in the training phase of the SVM. Moreover, whenever the l1 norm is optimized, tuning *C* implies the number, and the position, of the selected sensors: increasing *C* turns out to increase the number of selected sensors, and, in turn, vary the classification performances of the trained model. This is the pivotal point behind the automatic sensor selection. Indeed, if the l1 norm is minimized, the coefficient vector *w* turns out to be sparse after training, meaning that some of the features (i.e., some of the sensors readings) vanish, thus entailing a reduced number of selected sensors. Specifically, low values of *C* lead to *w* with more null elements, and thus fewer selected sensors. Conversely, whenever the l2 norm is optimized, *C* is related to the magnitude of *w* and *b*, since no sensor selection is performed throughout training. Indeed, l2 is chosen during the SVM training in the case in which sensors are manually selected a priori.

**Figure 3:**
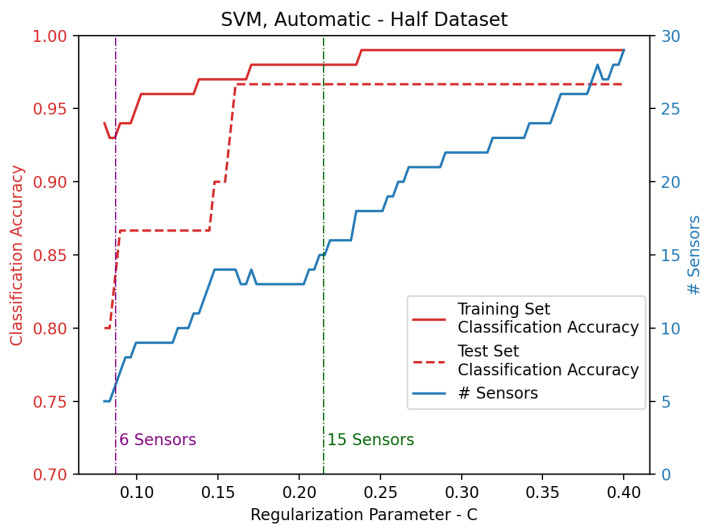
Classification accuracy for training and test set of the SVM developed on the upper part of the dataset. Sensors are automatically selected as a function of the regularization parameter *C*, where the vertical lines highlight the values for which 6 and 15 sensors are selected.

**Figure 4:**
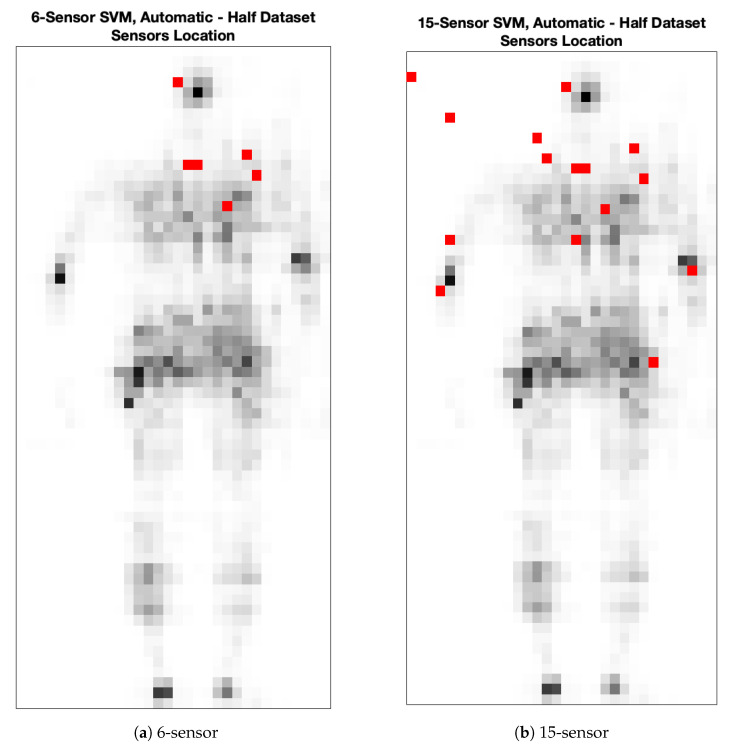
Automatically selected sensor locations for the SVM developed on the upper part of the dataset. Sensors are indicated by red dots, while gray-scale dots represent an example of pressure values acquired by the high-density sensor grid that occupies the whole rectangle. This shape is an example of pressure field measured by the original grid. Please notice that two contiguous sensors are selected on the chest zone.

Sensors of the upper part of the dataset are automatically selected according to the results of Figure 3. The performances of the SVM increase with *C*. Similarly, the number of input sensors becomes higher as *C* increases. This is reasonable, since the model is more capable of correctly estimating the dataset classes if more sensors are exploited. Then, two SVMs are selected, so that 6 and 15 sensors are, respectively, considered as input. Such conditions are in turn met when C=0.0869, giving 6 sensors, and C=0.2151, giving 15 sensors. Then, concerning the mean classification accuracy on the test set, the two SVMs provide 0.83 and 0.96, respectively, when 6 and 15 sensors are selected. Such sensors are also reported in Figure 4 for both the 6- and 15-sensor SVMs.

**Figure 5:**
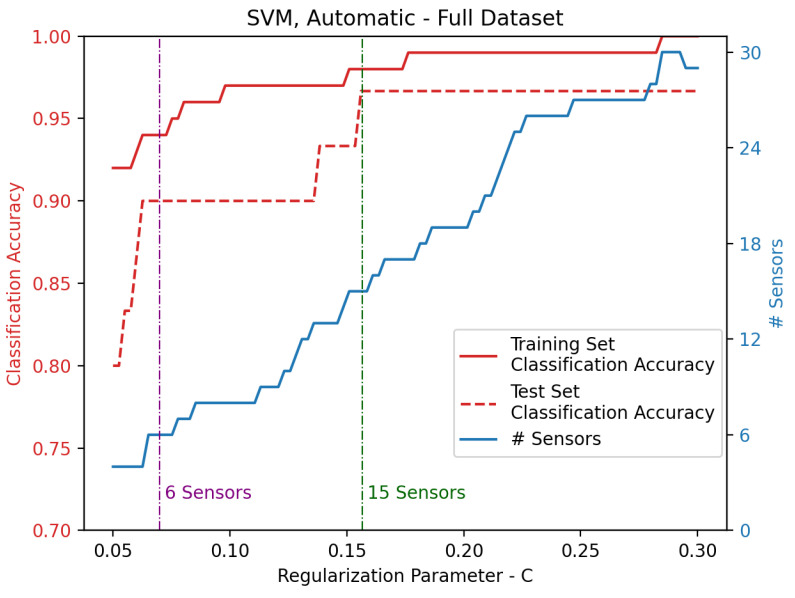
Classification accuracy for training and test set of the SVM developed on the whole dataset. Sensors are automatically selected in function of the regularization parameter *C*, where the vertical lines highlight the values for which 6 and 15 sensors are selected.

**Figure 6:**
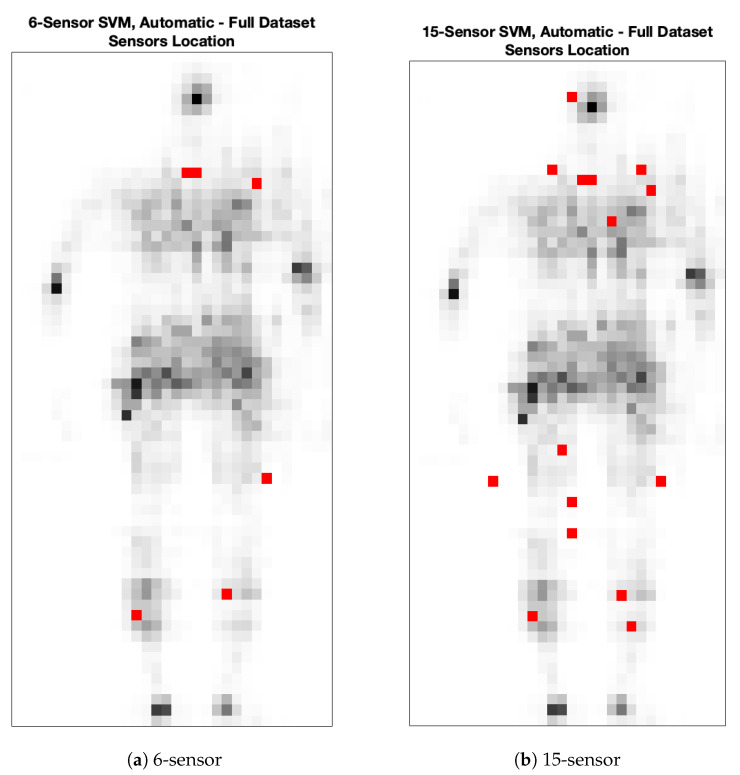
Automatically selected sensor locations for the SVM developed on the whole dataset. Sensors are indicated by red dots, while gray-scale dots represent an example of pressure values acquired by the high-density sensor grid that occupies the whole rectangle. This shape is an example of the pressure field measured by the original grid. Please notice that two contiguous sensors are selected on the chest zone.

Sensors of the whole dataset are automatically selected according to the results of Figure 5. The classification accuracy depends on the regularization parameter *C*, which in turn influences the number of selected sensors. Even in this case, two SVMs are selected, so that 6 and 15 sensors are respectively considered as input. Respectively, 6 sensors are selected for C=0.0700, giving 0.90 as mean classification accuracy on the test set, while 15 sensors are chosen for C=0.1567, providing 0.96 in terms of mean classification accuracy on the test set. Such sensors are also reported in Figure 6 for both the 6- and 15-sensor SVMs.

**Figure 7:**
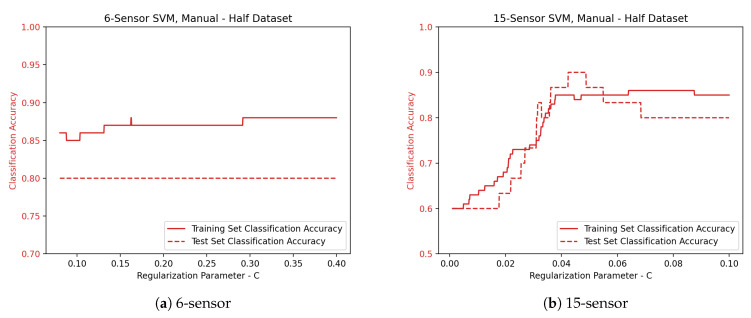
Classification accuracy for training and test set of the SVM developed on the upper part of the dataset, which takes as input the manually selected sensors.

The regularization parameter *C* influences the performances of the model even when sensors are manually selected. Specifically, Figure 7 displays the mean classification accuracy on both the training and test sets and for both the 6- and 15-sensor SVM, when the regularization parameter *C* is varied, whose spanning intervals were chosen aiming at maximizing performances while avoiding overfitting. The six-sensor SVM provides a constant mean classification accuracy on the test set (i.e., 0.73) for C≥0.1100, while the performances on the training set remain almost constant as *C* increases. This means that the model only accounting for six sensors manually chosen is not capable of properly learning the underlying pressure field sampled by the sensors in the mattress. Performances get better when 15 input sensors are considered: on the training set they are directly dependent on *C*, and the same behavior can be perceived on the test set up to C=0.0450. This means that considering those 15 sensors allows to develop an SVM capable of learning the pressure field, and that for C≥0.0485 overfitting arises. Therefore, if C=0.0450, that is the smallest regularization parameter ensuring the higher mean classification accuracy on the test set, the relative SVM provides a mean classification accuracy of 0.90.

## 5. Results and Discussion

**Figure 8:**
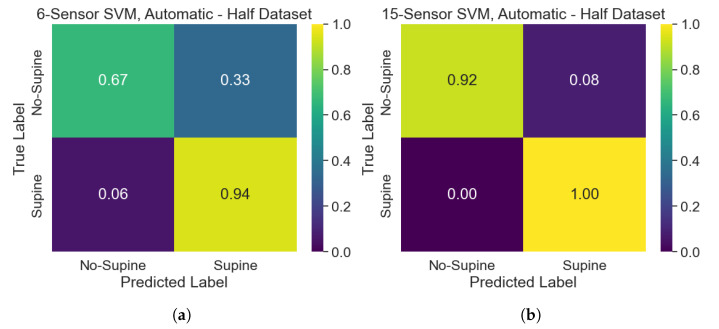
Confusion matrices, related to the test set, of the SVMs trained on the automatically selected sensors belonging to the upper part of the dataset: (**a**) 6-sensor SVM; (**b**) 15-sensor SVM.

**Figure 9:**
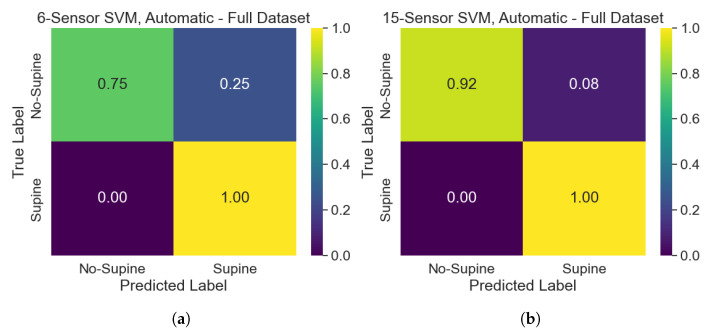
Confusion matrices, related to the test set, of the SVMs trained on the automatically selected sensors belonging to the whole dataset: (**a**) 6-sensor SVM; (**b**) 15-sensor SVM.

**Figure 10:**
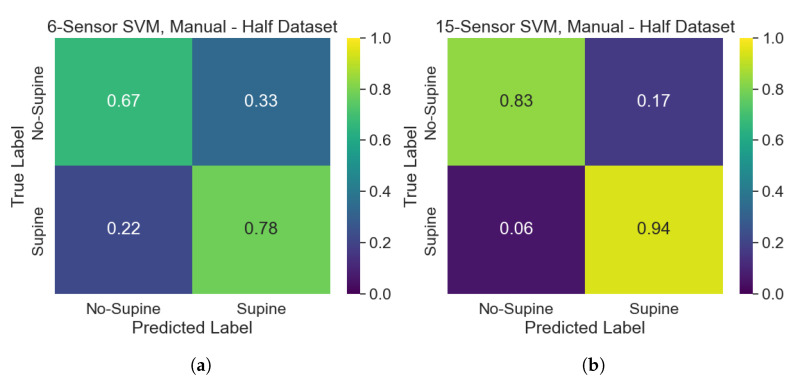
Confusion matrices, related to the test set, of the SVMs trained on the manually selected sensors: (**a**) 6-sensor SVM; (**b**) 15-sensor SVM.

The classification performances of the developed SVM models can be assessed and compared by making use of confusion matrices. The performance of the SVMs trained on the automatically selected sensors belonging to the upper part of the dataset on the test set can be assessed according to the confusion matrices of Figure 8. Overall, the model has better performances when 15 sensors are selected, since it tends to misclassify “No-Supine” postures when only 6 sensors are considered. Indeed, such a model suffers from a lack of specificity concerning the “No-Supine” class. Performances improve when the SVMs developed on the whole dataset are analyzed, as can be seen in Figure 9. Specifically, while the performances related to the 15-sensor SVM stay the same, the ones of the 6-sensor SVM get better, especially when “No-Supine” examples are classified, albeit a less remarked lack of specificity concerning such a class is nonetheless experienced. Conversely, the performances associated with the SVM models trained on the manually selected sensors are worse (see Figure 10): while the performances of the 15-sensor SVM can be viewed as good for a binary classifier, the ones related to the 6-sensor SVM are definitely worse, meaning that such sensors are not representative enough to properly distinguish between supine and non-supine postures.

**Figure 11:**
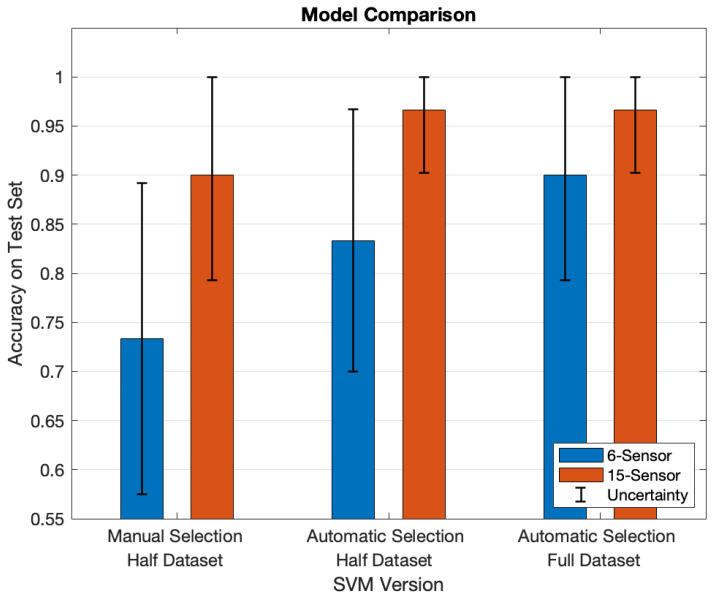
SVM performance comparison from the point of view of classification accuracy on the test set.

Finally, the models’ performances can be compared, as in Figure 11, where the associated uncertainties, related to a confidence interval of 95%, are reported as well. First of all, for a given number of input sensors, SVMs in which those sensors are automatically selected during training behave better than the SVM trained on a manually selected set of sensors. This hints at the fact that SVMs are capable of spotting underlying patterns, which are beneficial when these models solve a problem like this (i.e., classification and feature selection simultaneously). Specifically, such a property is more evident for models taking as input six sensors. Indeed, when they are automatically selected during training, the resulting SVMs strongly outperform the counterparts that are trained with the manually selected six sensors. On the other hand, this characteristic is milder when 15 sensors are chosen. Moreover, the higher correlation between the sensors belonging to the upper part of the mattress affects the performances only when six sensors are automatically chosen. Indeed, no performance degradation is perceived when 15 sensors are automatically selected, regardless of the fact that just the upper part of the mattress is exploited instead of its totality.

Concerning the type of selected ML model (i.e., the linear SVM), it was chosen in order to obtain a model that was as lightweight as possible in order to be run on a resource-constrained embedded device like the microcontrollers we considered (see Section 2). Indeed, from an implementation perspective, such a model boils down to a simple multiply–accumulate operation, once it is trained offline to find the optimal coefficient and bias (as it usually happens in standard embedded ML applications). Moreover, exploiting the linear SVM fulfills a twofold scope simultaneously: classification and sensor selection. Specifically, the latter is fundamental for the implementation of the system on a microcontroller, since acquiring 2048 pressure sensors at the same time is unfeasible on such platforms. Nonetheless, both tasks could have been carried out by resorting to other models (e.g., Least Absolute Shrinkage and Selection Operator (LASSO), Logistic Regression Classifier, etc.), but they would have been more involved when deployed on a microcontroller. Moreover, different models could select a different set of sensors and, alternatively, if trained with the ones selected by the linear SVM, an unfair comparison would occur.

Defining a gold standard in this type of application is extremely complicated, and it goes far beyond the scope of this work. Indeed, the topic covered is quite niche. However, other papers that deal with sleep posture identification with pressure sensors could be considered. In any case, this work can be compared with such works by limiting the scope to those exploiting the same dataset we adopted (i.e., [17]), in order to make a comparison as fair as possible. Nonetheless, this comparison must be carried out by bearing in mind that our goal is to have a system with rigid and well-defined constraints, which may be very different from those considered in other similar works.

Sleep posture classification was tackled in [18], where supine position was distinguished from left lateral and right lateral, meaning that a ternary classifier was set up, instead of a binary one as we are proposing. Similar results were obtained, but instead different ML models were exploited: a CNN, achieving a mean classification accuracy equal to the configuration accounting for 15 sensors (automatically selected from the full dataset) that we developed, and an RF behaving like the 6 sensor configuration (manually selected) that we put forth. In the same vein, [19,20,21] also proposed a CNN obtaining almost no misclassifications. However, those models required all of the sensor readings, since no sensor selection was performed. In the same fashion, [22] also made use of all of the sensor readings of the dataset, in order to train three ML models (i.e., a kNN, an NB, and an ANN). This means that such solutions are far from being implementable on the embedded devices we considered, also taking into account the computational burden required to run a CNN on a microcontroller. Another contribution devising an ANN is [23], where a Spiking Neural Network (SNN) achieving a mean accuracy of 92.40% was presented: despite the obtained results, this alternative is also not embedded-oriented, and needs all of the sensor readings to be trained. The same authors of [23] also investigated other techniques: in [24], they devised a particular CNN (achieving a mean accuracy of 95.32%) to be deployed on a single-board computer, rather than a microcontroller, and in [25] they converted a CNN into an SNN (obtaining a mean accuracy of 90.56%), without considering the option to deploy it on a microcontroller architecture. On the other hand, [26] presents a CNN achieving a mean accuracy of 96.77% (thus being comparable to the results we obtained) that was devised to be deployed on a Field-Programmable Gate Array (FPGA), rather than a microcontroller. Therefore, in the face of comparable results, it is preferable to opt for a linear SVM running on a microcontroller, since it is a far more lightweight model to train and run, because it relies on a reduced set of sensors, rather than a CNN trained on all of the sensors of the dataset.

## 6. Conclusions

In conclusion, our work proposes a novel approach to sleep posture detection by optimizing the configuration of a reduced pressure sensor array, employing an SVM-based sensor selection algorithm. This strategy enables the use of low-cost platforms for data acquisition and allows real-time classification directly on resource-constrained microcontrollers, conversely to traditional approaches requiring extensive sensor grids or costly computational resources [12,16]. The suitability of the solution of this work is highlighted by the obtained results in terms of mean classification accuracies: 0.90 and 0.96 for the configurations with 6 and 15 sensors, respectively, on the full dataset. When only the upper section of the dataset is exploited, accuracy for the 15-sensor setup remains stable at 0.96, while the 6-sensor configuration experiences only a minor decrease of 0.07.

Moreover, by processing raw data locally on embedded devices, the system mitigates data transmission demands, enhancing both security and privacy, thus ensuring two pivotal aspects where smart healthcare applications are concerned. Moreover, owing to the accuracy of the system, validated by the obtained results, our system holds promising implications for real-time health monitoring solutions, offering a scalable, cost-effective tool for the early detection and management of sleep-related health conditions.

Future works will address the current limitations of this study. In fact, like any other data-driven approach, our proposed solution suffers from the unavoidable process of collecting a representative and meaningful dataset to train the ML model. This translates into the fact that the model may lack sufficient generalization capabilities if it is supplied with data collected from postures related to subjects strongly differing, from the morphological perspective, from the ones involved during the gathering phase of the dataset we exploited [17]. However, the generalization capabilities of the model, as well as its robustness, can be significantly improved by performing a relatively short additional data acquisition phase aimed at tuning the model parameters. This may be needed in case the model is presented with data related to subjects strongly differing, from the morphological point of view, from the ones listed in Table 1. Moreover, with the availability of new data, we can make our model dynamic; the solution proposed in this manuscript is currently based on stable signal segments of a predefined duration, where no positional changes by the subject occur. By acquiring data with greater variability (i.e., position changes, limb movements, etc.), it will be possible to develop an enhanced version of the model that incorporates an artifact detector to identify instances of signal instability. This advancement will provide the model with dynamic capabilities, making it suitable for extensive, long-term monitoring. In the future, we plan to extend our work to other sleep and sleep-related pathologies, such as restless leg syndrome, parasomnias, and Parkinson’s disease. For these conditions, a more detailed and specific classification of movements may be required. This would include not only identifying various sleep positions (e.g., supine, non-supine, right, and left) but also detecting movement patterns and limb movements during sleep.

Additionally, a prototype of a mattress will be designed and developed, possibly also sampling other parameters (e.g., temperature, heart rate, saturation, movements, etc.), to help in carrying out a broad spectrum sleep monitoring system, which will be possibly capable of integrating with multiple platforms devoted to enhancing sleep quality (e.g., early diagnosing sleep bruxism devices [27]).

## Figures and Tables

**Table 1:** Detailed morphological information of the subjects involved during the dataset gathering phase.

Index	Age [Years]	Height [cm]	Body Mass [kg]
1	19	175	87
2	23	183	85
3	19	183	100
4	24	177	70
5	24	172	66
6	26	169	83
7	27	179	96
8	27	186	63
9	30	174	74
10	30	174	79
11	30	176	91
12	33	170	78
13	34	174	74

## Data Availability

The data used in this work are available at the following link: https://physionet.org/content/pmd/1.0.0/ (accessed on 19 November 2024).
